# First Multi-Facility Antimicrobial Surveillance in Japanese Hospital Wastewater Reveals Spatiotemporal Trends and Source-Specific Environmental Loads

**DOI:** 10.3390/antibiotics15010050

**Published:** 2026-01-03

**Authors:** Takashi Azuma, Ai Tsukada, Naoki Fujii, Miwa Katagiri, Itaru Nakamura, Hidefumi Shimizu, Keita Tatsuno, Manabu Watanabe, Norio Ohmagari, Nobuaki Matsunaga

**Affiliations:** 1 Department of Pharmaceutical Sciences, Osaka Medical and Pharmaceutical University, Takatsuki 569-1094, Japan; 2 Center for Infectious Disease Education and Research (CiDER), Osaka University, Suita 565-0871, Japan; 3 AMR Clinical Reference Center, National Center for Global Health and Medicine, Japan Institute for Health Security, Tokyo 162-8655, Japan; tsukada.ai@jihs.go.jp (A.T.); fujii.na@jihs.go.jp (N.F.); ohmagari.n@jihs.go.jp (N.O.); 4 Department of Surgery, Toho University Ohashi Medical Center, Tokyo 153-8515, Japan; katagiri@med.toho-u.ac.jp (M.K.); manabu@oha.toho-u.ac.jp (M.W.); 5 Department of Infection Prevention and Control, Tokyo Medical University Hospital, Tokyo 160-0023, Japan; task300@tokyo-med.ac.jp; 6 Department of Clinical Infectious Diseases, Tokyo Medical University Hospital, Tokyo 160-0023, Japan; 7 Department of Respiratory Medicine, Japan Community Healthcare Organization Tokyo Shinjuku Medical Center, Tokyo 162-8543, Japan; shimizu-hidefumi@shinjuku.jcho.go.jp; 8 Department of Infection Control and Prevention, Mitsui Memorial Hospital, Tokyo 101-8643, Japan; tatsuno-tky@umin.ac.jp; 9 Disease Control and Prevention Center, National Center for Global Health and Medicine, Japan Institute for Health Security, Tokyo 162-8655, Japan

**Keywords:** hospital wastewater, antimicrobials, wastewater monitoring survey, antimicrobial resistance (AMR), health care and the environment, high-throughput analysis

## Abstract

**Background**: Hospitals are recognized as point sources of antimicrobials in urban wastewater systems; however, comprehensive evaluations of their discharge profiles have not yet been conducted. **Methods**: This study presents a multi-site investigation of residual antimicrobial concentrations in effluents from five general hospitals and a commercial facility in the metropolitan area of Japan. Over a 12-week period (December 2023–March 2024), extensive sampling was conducted. Fifteen antimicrobials from multiple classes were quantified using high-throughput analysis. **Results**: The results revealed consistently higher concentrations in hospital effluents, particularly for levofloxacin, vancomycin, and ampicillin, than in non-clinical sites. Distinct facility-specific and temporal patterns suggest strong links between local prescribing practices and the effluent composition. Some compounds, such as clarithromycin and minocycline, showed dual contributions from both hospital and commercial sources. **Conclusions**: These findings highlight the need for source-targeted monitoring and antimicrobial pollution control strategies and provide a foundation for expanding surveillance efforts and informing environmental policies related to antimicrobial resistance (AMR).

## 1. Introduction

The widespread and increasing use of antimicrobials in clinical, veterinary, and community settings has prompted global concern regarding their environmental dissemination [1], particularly in urban wastewater systems [2]. Hospital effluents, in particular, represent a significant point source of pharmaceutical contaminants due to concentrated antimicrobial use and often limited on-site treatment infrastructure [3]. These discharges contribute not only to chemical pollution but also to the proliferation of antimicrobial resistance (AMR), which is recognized as one of the most pressing threats to global public health and environmental sustainability [4].

Although antimicrobial resistance (AMR) has historically been investigated primarily within clinical settings, accumulating evidence indicates that antimicrobials and antimicrobial-resistant bacteria are pervasive in aquatic environments, including rivers, lakes, and coastal waters [5]. Environmental AMR is now widely recognised not only as a driver of ecological disturbance but also as a significant threat to both human and animal health [6]. Potential risks encompass direct human exposure and infection through contact with contaminated waters [7], the emergence of novel resistant strains facilitated by horizontal gene transfer and genetic transformation in environmental matrices [8], and the amplification and dissemination of resistance within aquatic microbial communities, leading to enhanced disease transmission [9]. Taken together, these interconnected pathways underscore that AMR contamination in aquatic environments constitutes a largely underestimated yet potentially severe public health hazard [10].

The fate of antimicrobial residues discharged from healthcare facilities has received considerable attention over the past decade. Numerous studies have documented the occurrence of pharmaceuticals in municipal wastewater, surface waters, and even drinking water supplies, underscoring the challenges of conventional wastewater treatment systems in effectively removing micropollutants [11]. Among these sources, hospital wastewater has been identified as a critical contributor, particularly in urbanized regions, where centralized treatment plants often receive combined flows from diverse sources [12]. The concentrated nature of antimicrobial use in hospitals, often involving broad-spectrum agents and high-dose regimens, distinguishes hospital wastewater from typical domestic and industrial wastewaters.

Despite growing awareness, comprehensive and comparative evaluations of antimicrobial emissions across multiple hospitals within a single metropolitan context remain rare. Most existing studies have focused on single-facility assessments or employed low-resolution sampling strategies that may not capture the spatiotemporal variability inherent in pharmaceutical discharge [13]. Furthermore, the lack of empirical data linking facility-specific prescription practices, wastewater treatment characteristics, and antimicrobial profiles has hindered the development of targeted mitigation strategies [14]. The relative contributions of non-hospital sources, such as commercial facilities, pharmacies, and households, remain poorly characterized, although these sources may significantly influence ambient concentrations, particularly for antimicrobials commonly used in outpatient care [15].

In Japan, as in many other countries, there is an urgent need to develop robust monitoring frameworks that reflect the unique healthcare infrastructure, prescribing behaviors, and wastewater management practices [16]. Although several national-level studies have evaluated the occurrence of pharmaceuticals in surface waters and treatment plant influents, systematic investigations of residual antimicrobials in hospital wastewater remain limited [17]. Importantly, Japan’s universal health coverage system and aging population contribute to high antimicrobial consumption in both inpatient and outpatient settings, thereby amplifying the potential environmental burden [18]. To address these critical knowledge gaps, this study conducted a systematic and high-frequency monitoring campaign of antimicrobial concentrations in effluents from five hospitals and one commercial facility in an urban region of Japan. Sampling was carried out weekly from December 2023 to March 2024 for hospitals, and from March 2024 to February 2025 for commercial facilities, respectively. A suite of commonly prescribed antimicrobials representing different therapeutic classes was evaluated using high-throughput analytical systems, and facility-specific distribution patterns were characterized to identify the spatial and temporal trends.

This study had three objectives: (i) to characterize the spatial and temporal variability of antimicrobial concentrations in healthcare and commercial effluents; (ii) to delineate source-specific discharge profiles and identify high-load compounds; and (iii) to provide a robust empirical basis for developing targeted monitoring programs and antimicrobial pollution mitigation strategies. This study represents the first coordinated multi-site investigation of hospital wastewater contamination by antimicrobials in Japan and contributes novel insights relevant to the global discourse on AMR prevention and sustainable urban water resource management.

## 2. Materials and Methods

### 2.1. Chemicals and Reagents

A total of 17 antimicrobials were evaluated in five categories; *β*-lactam; ampicillin, benzylpenicillin, cefdinir, cefpodoxime, cefpodoxime proxetil, ceftiofur, new quinolones; ciprofloxacin, enrofloxacin, levofloxacin, macrolide; azithromycin, clarithromycin, tetracycline; chlortetracycline, doxycycline, minocycline, oxytetracycline, tetracycline, glycopeptide; vancomycin) were analyzed. This selection was based on previous reports of their concentration levels and frequencies of detection in hospital wastewater, WWTPs, and river water both in Japan and worldwide [19,20] and on the antimicrobial use in medical settings in Japan [21,22]. The physicochemical properties of each antibacterial are shown in Table 1. All analytical standards were of high purity (>98% purity) and were purchased from Cayman Chemical (Ann Arbor, MI, USA) and LKT Laboratories (St. Paul, MN, USA), and Santa Cruz Biotechnology, Inc. (Santa Cruz, CA, USA) and Tokyo Chemical Industry Co., Ltd. (Tokyo, Japan), and Toronto Research Chemicals, Inc. (Toronto, ON, Canada). Individual standard stock solutions (10 mg/L) were prepared in methanol, considering the purity of each reagent, and stored at −20 °C. All aqueous solutions were prepared using ultrapure water (18.2 MΩ·cm) provided by a Milli-Q purification system (MilliporeSigma, Watford, UK). LC-MS-grade methanol, acetone, formic acid, reagent-grade ascorbic acid, and hydrochloric acid were purchased from FUJIFILM Wako Pure Chemical Corporation (Osaka, Japan). 

### 2.2. Survey of Hospital Wastewater at Multiple Facilities in Urban Area of Japan

To investigate the occurrence and distribution of residual antimicrobials in hospital effluents, a series of systematic wastewater sampling campaigns was conducted at five major general hospitals located in densely populated urban areas of Japan. In these hospitals, various wastewater types generated as a result of hospital activities are directly discharged into the public sewage systems. It was impossible to quantify the daily outflow of wastewater because of the lack of any system for regular measurements. Sampling was carried out on nine separate occasions between 19 December 2023, and 5 March 2024. These hospitals were selected based on their scale and regional centrality. For comparative purposes, wastewater from a commercial facility was monitored during the same period. This commercial facility is a large-scale, comprehensive shopping mall located in the densely populated urban area where the hospital targeted in this study is investigated. The wastewater from the commercial facility is discharged directly into the public sewage system.

Because of the impracticality of installing automatic composite samplers at the selected sites, grab sampling was used as an alternative method. Wastewater samples were manually collected at fixed intervals following previously established protocols [24]. Immediately after collection, each wastewater sample was filtered using TPP Rapid Filtermax vacuum filtration systems (TPP, Trasadingen, Switzerland) into 150 mL bottles, as described previously [19]. The filtered samples were promptly transported to the laboratory in insulated cooler boxes and frozen at −20 °C to prevent degradation. Subsequently, samples were shipped on dry ice to the authors’ laboratory, where they were thawed at 4 °C in complete darkness prior to analysis.

### 2.3. Analytical Procedures for Antimicrobials in the Wastewater Based on the High-Throughput Analysis

The analysis of antimicrobials in wastewater was conducted using a combination of automated pipetting equipped with solid-phase extraction (SPE) systems (Andrew + Pipetting Robot (Waters Corp., Milford, MA, USA) and Extraction + Base Kit WITH Gripper (Waters Corp.)) and ultra-performance liquid chromatography–tandem mass spectrometry (UPLC–MS/MS), as previously described [23]. Wastewater (2.5 mL) was passed through OASIS HLB 96-well SPE plates with a 30 mg solid-phase carrier (Waters Corp.) that was preconditioned with 500 μL of methanol and 500 μL of Milli-Q water adjusted to pH 3 with 1 N hydrochloric acid. All cartridges were cleaned by washing with 500 μL of Milli-Q water pre-adjusted to pH 3 and dried using a vacuum pump. The adsorbed antimicrobials were eluted with 500 μL of a 1:1 (*v*/*v*) mixture of acetone and methanol and evaporated to dryness under a gentle stream of N_2_ gas at 37 °C. The residue was solubilized in 100 μL of a 90:10 (*v*/*v*) mixture of 0.1% formic acid in methanol. Finally, 10 μL of this solution was analyzed using a UPLC–MS/MS fitted with a column (2.1 mm × 100 mm, 1.7 μm) UPLC BEH C_18_ column (Waters Corp.) coupled to a tandem quadrupole mass spectrometer (Waters Corp.).

### 2.4. Method Validation

Six-point standard calibration curves were constructed for quantification ranging from 0.5 to 200 ng/mL in a 90:10 (*v*/*v*) mixture of 0.1% formic acid in methanol. Individual linear calibration curves for each compound were obtained in the concentration range 0.5 of 200 ng/mL (*r*^2^ > 0.99) using a weighting factor of 1/x. Instrument control and quantification were performed using Mass Lynx 4.1 software (Waters Corp.).

Quantification involved subtracting the blank data from the corresponding data obtained from the spiked sample solutions to account for the matrix effects and losses during sample extraction [25]. Similarly, the recovery rates were calculated from the deviations between spiked and standard calibration data [26]. The limits of detection (LODs) and limits of quantification (LOQs) for the water samples were calculated based on the concentrations at signal-to-noise ratios of 3 and 10, respectively, according to methods applied to pharmaceuticals in river water and wastewater samples [27]. These profiles are shown in Table S1 and are generally similar to those previously reported for pharmaceuticals in river water and wastewater samples [28].

### 2.5. Statistical Analysis

The basic data for the tested traits were analyzed using Microsoft Excel (Microsoft 365) and presented as mean values and their individual standard deviations, while other statistical analyses and graphics were performed using R software (version 4.5.0) (https://www.R-project.org/). A paired *t*-test was performed to assess the differences between the water samples, with statistical significance set at *p* < 0.05. Violin and density plot analyses were performed using the “ggplot2” package [29] in R (version 4.5.0). All visualizations adhered to standardized formatting and scaling procedures to ensure consistency across the comparative datasets.

## 3. Results and Discussion

### 3.1. Distribution of Antimicrobial Concentrations in Wastewater from Hospitals and a Commercial Facility

**Figure 1:**
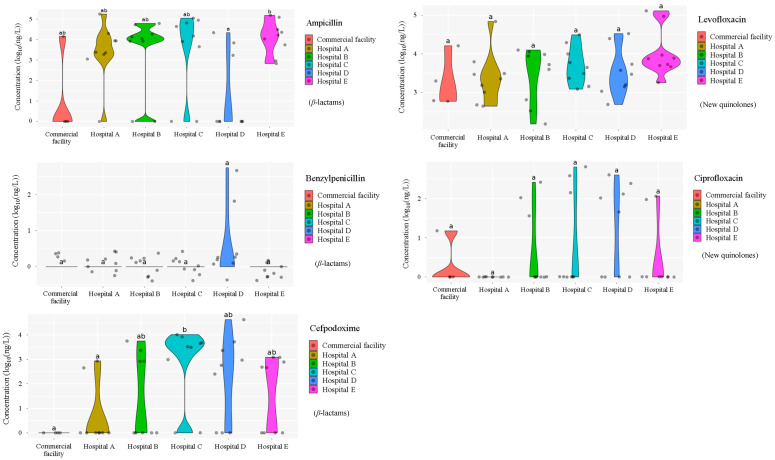

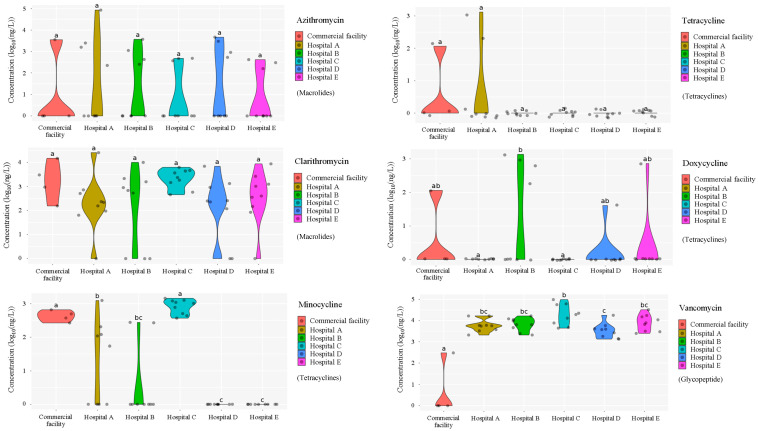
Distribution of concentrations of antimicrobials in five different hospital wastewater (Hospital A–E) effluent and wastewater from commercial facility investigated in the urban regions of Japan. Different characters within the violin plot indicate significant differences between groups (*p* < 0.05).

The concentrations of fifteen antimicrobials detected in wastewater from five hospitals (Hospitals A–E) and one commercial facility are summarized in Figure 1. Eleven antimicrobials were detected in hospital wastewater and eleven in commercial facility wastewater, with marked variability in both detection frequency and concentration among sites. These spatial differences indicate heterogeneity in antimicrobial usage patterns, wastewater handling practices, and potential removal efficiencies [30].

#### 3.1.1. *β*-Lactams

Ampicillin concentrations were significantly higher in hospital effluents than in the commercial facility, reaching maxima of 176 μg/L (Hospital A) and 154 μg/L (Hospital E). Several samples from Hospitals B–D exceeded 4 log_10_ (>10 μg/L), whereas only sporadic, lower concentrations were observed in the commercial facility. These results indicate that hospitals are the primary contributors to ampicillin loads in wastewater, consistent with its widespread clinical use as a first-line penicillin-class antimicrobial [31].

Benzylpenicillin was largely undetected across all sites, with only isolated detections in Hospital D, suggesting limited use and/or rapid degradation, potentially linked to hospital-specific prescribing practices.

Cefpodoxime was intermittently detected in hospital effluents at concentrations ranging from 2.5 to 5.5 log_10_ (hundreds of ng/L to hundreds of μg/L), with notably elevated levels at Hospitals C and D, reaching 154 μg/L and 43 μg/L, respectively. In contrast, cefpodoxime was not detected in wastewater from the commercial facility, and concentrations differed significantly between hospital and commercial facility wastewater (*p* < 0.05).

#### 3.1.2. New Quinolones

Ciprofloxacin was intermittently detected in both hospital and commercial facility wastewater, with concentrations of 35–644 ng/L and 14 ng/L, respectively. Although concentrations tended to be higher in hospital effluents, detection frequencies were lower than those of clarithromycin, levofloxacin, and vancomycin, indicating more sporadic usage patterns [32].

Levofloxacin was consistently detected (100% detection frequency) at elevated concentrations in effluents from all hospitals (Hospitals A–E), spanning a broad range of 2.2–5.1 log_10_ (tens of ng/L to tens of μg/L). In commercial facility wastewater, levofloxacin was also detected at comparable concentrations (2.2–4.2 log_10_), and no significant difference (*p* < 0.05) was observed between hospital and commercial facility wastewater. The ubiquitous detection of levofloxacin across both wastewater types suggests combined clinical and community contributions, consistent with its widespread prescription in both inpatient and outpatient settings [33,34].

#### 3.1.3. Macrolides

Clarithromycin was consistently detected at relatively high concentrations in all hospitals, spanning 1.9–4.4 log_10_ (tens of ng/L to tens of μg/L), and occurred at comparable levels in commercial facility wastewater. This widespread presence likely reflects its broad-spectrum activity and extensive use in both inpatient and outpatient settings [35], consistent with national prescription statistics in Japan [21].

Azithromycin was detected in wastewater from all hospitals and the commercial facility, with detection frequencies of 30–44% (hospitals) and 25% (commercial facility). Concentrations exhibited wide variability (2.2–4.9 log_10_), reflecting episodic, high-dose prescribing associated with acute infections [36] and temporal fluctuations inherent to grab sampling [37].

#### 3.1.4. Tetracyclines

Doxycycline was detected at elevated concentrations in Hospitals B, D, and E, with substantial inter-sample variability. Minocycline was intermittently detected in Hospitals A–C (53 ng/L–1.5 μg/L) and was consistently detected (100% frequency) in Hospital C. Tetracycline was detected exclusively in Hospital A, at concentrations of 272 ng/L to 1.3 μg/L. Comparable detection patterns were observed in commercial facility wastewater, albeit at lower and less variable concentrations (doxycycline: 113 ng/L; minocycline: 265–658 ng/L; tetracycline: 114 ng/L). These localized and heterogeneous distributions likely reflect the more specialized clinical indications of tetracycline-class antimicrobials, potentially linked to specific hospital departments or prescription practices [38].

#### 3.1.5. Glycopeptide

Vancomycin was detected infrequently during the study (25% detection frequency). When detected, concentrations in hospital effluents were comparable across sites, spanning a broad range of 3.1–5.0 log_10_ (several μg/L to several hundred μg/L). In commercial facility wastewater, vancomycin was detected only at low levels, around 2.5 log_10_ (hundreds of ng/L). Consequently, vancomycin concentrations differed significantly between hospital and commercial facility wastewater (*p* < 0.05). Given its clinical role in treating antimicrobial-resistant infections, these patterns likely reflect targeted use in inpatient and intensive care settings [39,40]. The near absence of vancomycin in commercial facility wastewater further indicates that hospitals are the primary point sources of this antimicrobial.

Cefdinir, ceftiofur, enrofloxacin, and chlortetracycline were not detected at any site, likely reflecting limited human usage or preferential veterinary application, particularly for ceftiofur and enrofloxacin [41]. Conversely, the absence of cefdinir and chlortetracycline was likely due to low usage or degradation caused by instability in environmental water at the time of the study period. These results are generally consistent with those of surveys of sewage treatment plants and river water in Japan [42].

Distinct antimicrobial concentration profiles between hospitals and the commercial facility highlight differences in usage intensity, therapeutic practices, and point-source discharge characteristics [43]. Broad-spectrum antimicrobials, including clarithromycin, levofloxacin, and vancomycin, were detected in nearly all hospital samples, consistent with their high prescription rates and previous wastewater surveillance studies [23]. In contrast, the limited detection of benzylpenicillin and cefpodoxime likely reflects lower usage frequencies and/or enhanced degradation prior to discharge, potentially associated with hospital-specific wastewater management practices [44]. The absence of most antimicrobials in commercial facility wastewater further supports the conclusion that hospitals represent dominant point sources of antimicrobial residues in urban wastewater systems [45].

Elevated concentrations of vancomycin, doxycycline, and ciprofloxacin in hospital effluents suggest targeted use in inpatient care and surgical settings, where antimicrobial-resistant infections are more prevalent. Conversely, the detection of levofloxacin in both hospital and commercial effluents indicates widespread outpatient use and substantial community-derived contributions. Inter-hospital variability in antimicrobial detection patterns likely arises from differences in patient demographics, therapeutic preferences, formulary composition, and the presence or absence of on-site pretreatment systems [46]. These site-specific characteristics underscore the need for facility-tailored monitoring frameworks and targeted mitigation strategies aligned with antimicrobial usage profiles [47].

### 3.2. Temporal Dynamics of Antimicrobials in Hospital Wastewater

**Figure 2:**
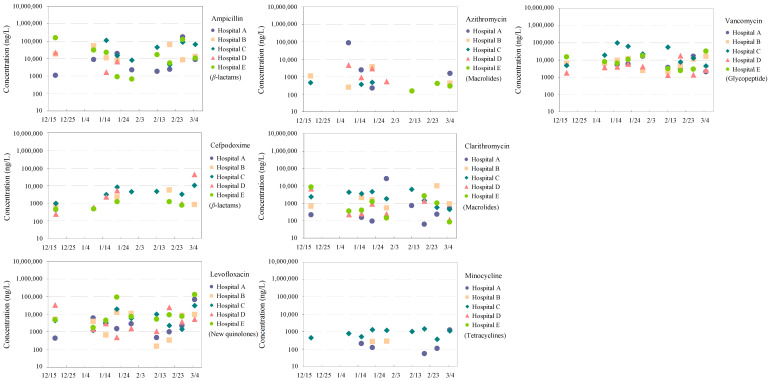
Temporal trends in detected concentrations (ng/L) of seven representative antimicrobials (ampicillin, cefpodoxime, levofloxacin, azithromycin, clarithromycin, minocycline, and vancomycin) in wastewater from five hospitals (Hospitals A–E) over a monitoring period.

Temporal trends in antimicrobial concentrations across five hospitals (Hospitals A–E) are shown in Figure 2, with corresponding mean concentrations, including those from a commercial facility, summarized in Table 2. The results demonstrate marked facility-specific patterns and temporal variability, reflecting heterogeneity in clinical practices, patient populations, and on-site wastewater treatment configurations [48].

#### 3.2.1. *β*-Lactams

Ampicillin was consistently detected in all hospital effluents at high concentrations throughout the monitoring period. The highest mean levels were observed in Hospitals C (48 μg/L ± 42 μg/L) and E (41 μg/L ± 57 μg/L), reflecting its extensive use as a first-line broad-spectrum β-lactam in both inpatient and outpatient care [49]. A single elevated ampicillin concentration (14 μg/L) was also detected in the commercial facility, likely attributable to outpatient prescriptions or short-term concentration spikes captured by grab sampling [50].

By contrast, benzylpenicillin was detected exclusively in Hospital D at an exceptionally high concentration (158 μg/L). This isolated occurrence likely reflects targeted administration in specialized units (e.g., obstetrics or infectious disease wards) [51], illustrating how episodic clinical use can disproportionately shape wastewater profiles.

Cefpodoxime was detected in all hospital effluents, with the highest mean concentrations in Hospital D (9 μg/L ± 17 μg/L) and Hospital C (5 μg/L ± 3 μg/L), suggesting frequent prescription and/or limited removal by on-site wastewater treatment systems. In contrast, cefpodoxime proxetil was detected only in the commercial facility (20 ng/L), indicating a predominantly non-hospital origin, such as outpatient use or community-derived wastewater.

Despite the rapid degradation of *β*-lactams in aquatic environments [52], their high concentrations in raw effluents indicate substantial short-term contributions to environmental antimicrobial loading prior to degradation.

#### 3.2.2. New Quinolones

Ciprofloxacin and levofloxacin were widely detected across hospital effluents. Levofloxacin reached particularly high concentrations in Hospital E (29 μg/L ± 48 μg/L) and showed moderate yet stable levels in Hospitals B and C, consistent with its extensive use in acute and chronic treatments, especially for respiratory and urinary tract infections. Ciprofloxacin exhibited its highest mean concentration in Hospital C (389 ng/L ± 251 ng/L) and was detected in four of the five hospitals. Low-level detection in the commercial facility (14 ng/L) suggests minor but non-negligible community contributions.

These patterns are consistent with the high environmental persistence of fluoroquinolones and their extensive prescription in clinical settings [53].

#### 3.2.3. Macrolides

Azithromycin exhibited episodic peaks in Hospital A, reaching 23 μg/L ± 43 μg/L, whereas concentrations in other hospitals and the commercial facility remained low (3.5 ng/L). Such transient elevations likely reflect outbreak-driven prescription patterns, particularly during respiratory infection seasons or pandemics. Clarithromycin showed comparatively stable concentrations across facilities, with elevated levels in Hospital A (3 μg/L ± 9 μg/L) and the commercial facility (5 μg/L ± 7 μg/L), indicating combined hospital and non-hospital inputs. Its frequent detection reflects its broad-spectrum activity and routine use for both hospital- and community-acquired infections.

The presence of macrolides in both healthcare and commercial effluents highlights challenges in source attribution and underscores shared responsibility for pharmaceutical emission management [54].

#### 3.2.4. Tetracyclines

Tetracyclines exhibited sporadic and highly site-specific detection patterns. Doxycycline was detected only in Hospitals B (763 ng/L ± 497 ng/L) and E (723 ng/L), indicating localized clinical use. Minocycline reached its highest concentrations in Hospital C (905 ng/L ± 398 ng/L) and was also detected in the commercial facility (447 ng/L ± 168 ng/L), potentially reflecting use in dermatological or dental treatments and availability through non-prescription channels. Tetracycline itself was detected exclusively in Hospital A (795 ng/L ± 739 ng/L) and the commercial facility (114 ng/L), suggesting possible inputs from non-prescription or veterinary-related sources.

#### 3.2.5. Glycopeptide

Vancomycin was consistently detected at elevated concentrations in all hospitals, with the highest levels observed in Hospital C (32 μg/L ± 33 μg/L). This persistent presence reflects its critical role in treating multidrug-resistant infections in inpatient care [40]. The markedly lower concentration in the commercial facility (302 ng/L) further supports its predominantly hospital-based origin.

Facility-specific antimicrobial profiles reflect complex interactions among clinical practices, therapeutic specialization, and local infrastructure. Persistently high concentrations of ampicillin and vancomycin across hospitals underscore their central roles in empirical and advanced infection management. The isolated detection of benzylpenicillin in Hospital D and cefpodoxime proxetil in the commercial facility emphasizes the importance of high temporal and sectoral resolution in wastewater surveillance.

The widespread detection of fluoroquinolones is consistent with their resistance to degradation and broad-spectrum clinical application. Similarly, the distribution of macrolides across hospital and non-hospital sources highlights the need for surveillance frameworks extending beyond institutional boundaries.

Overall, these findings underscore the necessity of integrated, source-specific antimicrobial monitoring strategies [55]. While hospitals remain major emission points, non-hospital sources, including outpatient clinics and community pharmacies, must also be incorporated into surveillance programs. Effective mitigation requires consideration of compound-specific physicochemical properties, degradation behavior, and usage patterns to reduce antimicrobial loads entering municipal wastewater systems and ultimately limit the spread of antimicrobial resistance [56].

### 3.3. Temporal Variations and Distribution Patterns of Antimicrobials in Hospital and Commercial Wastewaters

**Figure 3:**
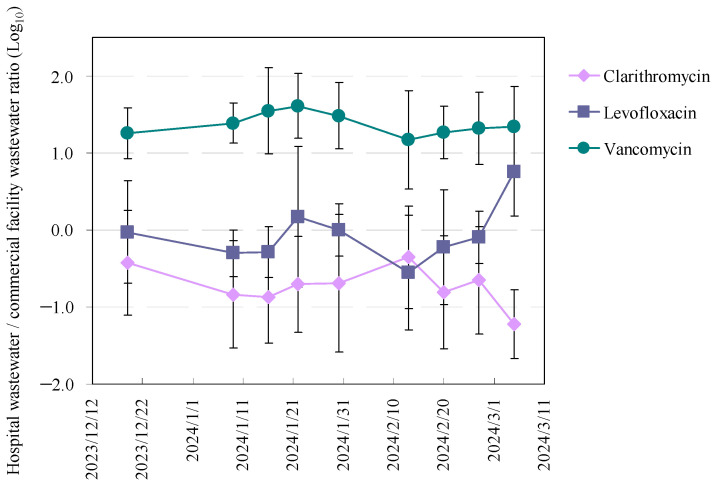
Temporal variation in the concentration ratio (hospital wastewater/commercial facility wastewater) for three representative antimicrobials (clarithromycin, levofloxacin, and vancomycin) over the sampling period from December 2023 to March 2024. The horizontal axis shows the passage of time during the drainage sampling period.

Finally, we examined the temporal variability and distribution patterns of antimicrobials in hospital and commercial wastewater, focusing on three frequently detected antibiotics: clarithromycin, levofloxacin, and vancomycin. The temporal dynamics of their concentrations in hospital wastewater relative to commercial effluents, expressed as log_10_ hospital-to-commercial concentration ratios, are shown in Figure 3, with a detailed statistical summary provided in Table S2.

For clarithromycin and levofloxacin, the ratios were predominantly negative throughout the monitoring period, indicating higher concentrations in commercial wastewater than in hospital effluent. The log_10_ ratios ranged from −1.9 to 0.7 for clarithromycin (mean: −0.7 ± 0.3) and from −1.5 to 1.3 for levofloxacin (mean: −0.1 ± 0.4). Both compounds exhibited substantially greater temporal variability than vancomycin. Intermittent positive ratios observed for levofloxacin indicate episodic increases in hospital discharges, likely reflecting its widespread use in both inpatient and outpatient clinical settings [57]. This interpretation is consistent with previous studies identifying macrolides and fluoroquinolones as commonly detected antimicrobials in hospital wastewater due to broad prescription practices [58,59].

In contrast, vancomycin exhibited consistently elevated hospital-to-commercial ratios, with log_10_ values ranging from 0.6 to 2.5 (mean: 1.4 ± 0.1), corresponding to hospital concentrations approximately 10–300 times higher than those in commercial wastewater. This pattern clearly identifies healthcare facilities as the dominant source of vancomycin emissions. The sporadic detection in commercial wastewater is likely attributable to excretion by patients undergoing home-based antimicrobial therapy. Temporal fluctuations in these ratios may reflect variations in patient load, treatment regimens, or institutional antimicrobial stewardship practices [60].

These time-resolved variations in antimicrobial emissions likely reflect seasonal prescribing trends, fluctuations in hospital admissions, and changes in infectious disease prevalence. Collectively, these findings highlight the necessity of longitudinal monitoring to accurately characterise pharmaceutical loading dynamics in healthcare-associated wastewater systems.

**Figure 4:**
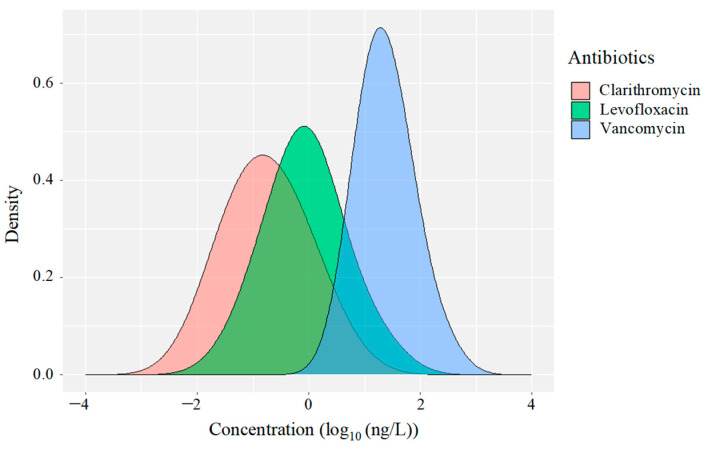
Density distribution of the concentrations (log_10_ ng/L) of clarithromycin, levofloxacin, and vancomycin in hospital and commercial facility wastewaters. The horizontal axis shows the log_10_ value of the detected antimicrobial concentration.

Figure 4 presents the probability density functions of the log10 hospital-to-commercial concentration ratios. Clarithromycin and levofloxacin showed broad, flattened distributions centred near or below zero, indicative of heterogeneous and episodic release patterns. Such dispersion suggests that peak concentrations arise from transient discharge events, potentially driven by facility-specific practices, sudden prescription surges, or treatment heterogeneity [50]. These patterns imply substantial variability in usage across healthcare and community sectors, supporting previous observations of frequent prescription in both hospital and outpatient settings [61]. In contrast, vancomycin exhibited a sharp, unimodal peak centred at approximately 2 log_10_, indicating consistently high and hospital-dominated discharge. This narrow distribution reflects its restricted clinical use, in stark contrast to the diffuse profiles of the other antimicrobials, and is consistent with observations from hospital-dominated sewage catchments [62].

The presence of heavy tails in the density distributions of all three compounds indicates the occurrence of high-intensity discharge events, potentially associated with mass antimicrobial administration or sudden patient surges. These results underscore the importance of high-resolution, high-frequency sampling strategies to capture dynamic and transient emission events [63,64]. A detailed understanding of these patterns is critical for developing targeted mitigation strategies that consider compound-specific pharmacokinetics, usage behaviours, and excretion pathways [65].

Notably, this study demonstrates a novel application of wastewater-based epidemiology (WBE) to resolve sector-specific differences in antimicrobial emissions [66]. By integrating concentration ratios, temporal dynamics, and distributional characteristics, we provide empirical evidence that WBE can effectively distinguish healthcare-derived from commercial antimicrobial sources. The distinct signatures of clarithromycin, levofloxacin, and vancomycin highlight the value of spatially and temporally resolved WBE for informed surveillance and targeted management strategies [67].

A comprehensive assessment of medical wastewater across human, societal, and environmental interfaces is essential for mitigating health risks associated with antimicrobial-resistant bacteria and related contaminants. This perspective emphasises the interconnectedness of healthcare practices and environmental sustainability, particularly in balancing modern medical care with long-term ecological resilience [68]. Importantly, this study provides the first nationwide dataset on residual antimicrobials in hospital effluents across Japan, establishing a critical baseline for evaluating AMR-related environmental contamination. The application of high-throughput analytical techniques further enables an efficient and scalable framework for nationwide monitoring of antimicrobial pollution [69].

Further collaboration with hospitals, local governments, and environmental agencies is required to expand monitoring networks and generate higher-resolution datasets. In parallel, systematic assessments of the ecological and human health risks posed by residual antimicrobials in aquatic environments are urgently needed to inform evidence-based risk management and regulatory frameworks [70]. Moreover, strengthening dialogue and technological collaboration among stakeholders, including policymakers and public health authorities, will be critical for translating these findings into practice and advancing innovations in antimicrobial pollution control [71].

## 4. Limitations

The limitations of this study are as follows: The first is the constraints on data interpretation. Prior to study initiation, all participating hospitals required the exclusion of any information that could enable institutional identification, including regional location, bed capacity, prescription profiles, and wastewater flow volumes. All reported data therefore fully comply with research ethics and confidentiality agreements. Consequently, direct analyses linking antimicrobial occurrence to hospital-specific clinical practices or facility characteristics were not feasible in the present study. Future multi-center investigations conducted under harmonized data-sharing frameworks, at both national and international scales, are expected to expand the available evidence and enable more detailed comparative assessments.

Second, the antimicrobials quantified in this study represent the parent active pharmaceutical ingredients (APIs) themselves. Previous studies have demonstrated that antimicrobial concentrations in wastewater and receiving waters generally decline over time as a result of biodegradation, hydrolysis, and other transformation processes [72,73]. In contrast, the wastewater examined here originates directly from hospitals and commercial facilities and is discharged into public sewer systems via toilets and sinks within minutes of release, following only brief residence times in facility-level drainage pipes. As a result, these samples closely reflect primary antimicrobial inputs associated with human excretion and disposal, with minimal alteration by in-sewer transformation processes. Investigating the behavior and fate of antimicrobials in wastewater and environmental waters is essential for understanding their transport, transformation, and persistence in aquatic systems. However, accurate and comprehensive quantification remains technically challenging, and significant international efforts are ongoing to improve analytical techniques and detection platforms. One of the most critical limitations is the scarcity of reference standards for antibiotic metabolites, which are indispensable for robust identification and quantification yet are largely unavailable from commercial suppliers or pharmaceutical manufacturers [74,75]. As a consequence, many current assessments rely on qualitative or indirect evaluations of antimicrobial transformation products. Addressing these methodological constraints represents a key priority and an important direction for future research in environmental antimicrobial studies. Our results support the need for further conclusive research that considers experimental, technical, and regional customs, bias, and unknown factors.

## 5. Conclusions

This study provides the first multi-site, high-resolution assessment of antimicrobial residues in hospital and commercial wastewater effluents in urban Japan, thereby clarifying the magnitude and variability of pharmaceutical contamination at the source level. Systematic weekly monitoring across five hospitals and one commercial facility demonstrated consistently elevated concentrations of clinically relevant antimicrobials, including ampicillin, levofloxacin, and vancomycin, in hospital effluents compared with non-clinical wastewater. These results clearly identify healthcare facilities as major point sources of antimicrobial discharge within urban wastewater systems. Marked temporal variability in antimicrobial concentrations was observed, reflecting differences in clinical activity, prescription practices, and facility-specific operational conditions. The pronounced heterogeneity among hospitals, including compound-specific discharge signatures, underscores the strong influence of localized medical practices, therapeutic focus, and on-site wastewater management capacity on antimicrobial emissions.

These findings highlight that uniform mitigation strategies are insufficient to address antimicrobial pollution originating from healthcare facilities. Source-specific interventions, including strengthened antimicrobial stewardship, targeted upgrades of hospital wastewater pretreatment systems, and facility-level high-resolution monitoring, are essential to effectively reduce antimicrobial loads and mitigate environmental AMR risks. The analytical framework established in this study provides a scalable basis for broader surveillance programs at regional and national levels. Future research should integrate downstream environmental fate assessments, resistance gene dynamics, and treatment performance evaluations to better quantify the full impact of hospital-derived antimicrobials. Overall, by explicitly linking clinical activities with environmental emissions, this study offers a robust scientific foundation for evidence-based policy development, risk management, and sustainable urban water governance addressing AMR-related pollution.

## Figures and Tables

**Table 1:** Chemical structures and physicochemical properties of the target antimicrobials examined [23].

Classification	CAS Registry Number	Antimicrobials	Molecular Formula	Molecular Mass (g/mol)	Structure	p*K*_a_	Log*P*
*β*-lactams	69-53-4	Ampicillin	C_16_H_19_N_3_O_4_S	349.4	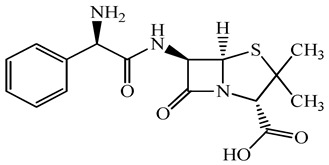	2.4	1.4
	61-33-6	Benzylpenicillin	C_16_H_18_N_2_O_4_S	334.4	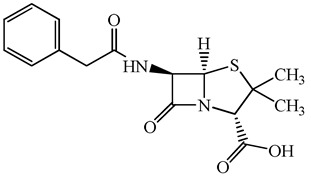	2.5	1.7
	91832-40-5	Cefdinir	C_14_H_13_N_5_O_5_S_2_	395.4	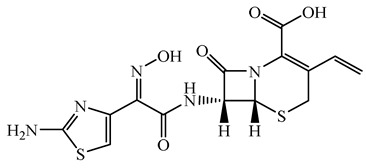	2.8	−1.8
	80210-62-4	Cefpodoxime	C_17_H_19_N_5_O_6_S_2_	453.5	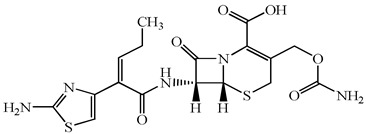	2.8 (Acid) 1.7 (Base)	0.4
	87239-81-4	Cefpodoxime proxetil	C_21_H_27_N_5_O_9_S_2_	557.6	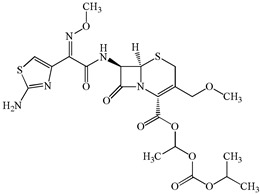	8.1 (Acid) 1.7 (Base)	2.9
	80370-57-6	Ceftiofur	C_19_H_17_N_5_O_7_S_3_	523.6	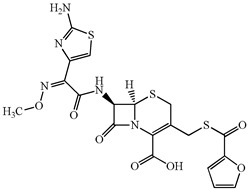	2.6	1.7
New quinolones	85721-33-1	Ciprofloxacin	C_17_H_18_FN_3_O_3_	331.3	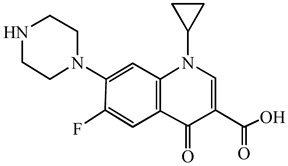	6.0	1.3
	93106-60-6	Enrofloxacin	C_19_H_22_FN_3_O_3_	359.4	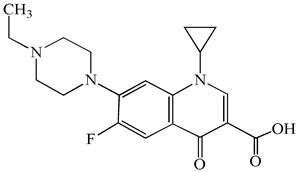	6.4	2.3
	100986-85-4	Levofloxacin	C_18_H_20_FN_3_O_4_	361.4	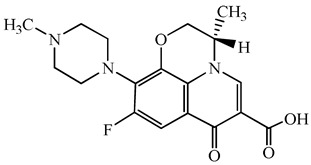	5.2	1.6
Macrolides	83905-01-5	Azithromycin	C_38_H_72_N_2_O_12_	749.0	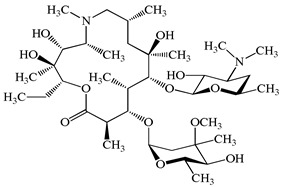	13.3	3.3
	81103-11-9	Clarithromycin	C_38_H_69_NO_13_	748.0	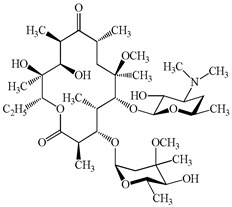	13.1	3.2
Tetracyclines	57-62-5	Chlortetracycline	C_22_H_23_ClN_2_O_8_	478.9	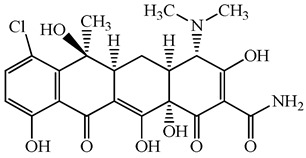	4.5	4.8
	564-25-0	Doxycycline	C_22_H_24_N_2_O_8_	444.4	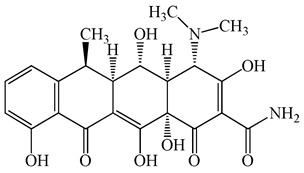	4.5 (Acid) 10.8 (Base)	1.8
	10118-90-8	Minocycline	C_23_H_27_N_3_O_7_	457.5	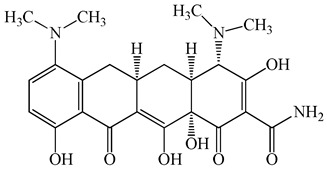	4.5 (Acid) 11.1 (Base)	2.2
	79-57-2	Oxytetracycline	C_22_H_24_N_2_O_9_	460.4	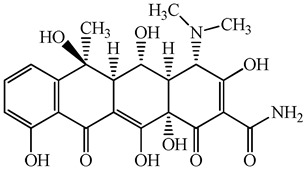	4.5	−1.5
	60-54-8	Tetracycline	C_22_H_24_N_2_O_8_	444.4	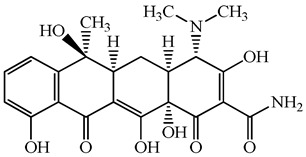	4.5	−1.5
Glycopeptide	1404-90-6	Vancomycin	C_66_H_75_Cl_2_N_9_O_24_	1449.3	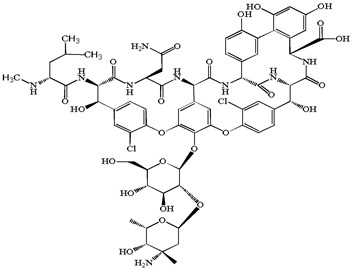	3.0	−1.4

CAS: chemical abstracts service; *pK*_a_: acid dissociation constant; Log*P*: octanol-water partition coefficients.

**Table 2:** Distribution of detected antimicrobial concentrations in wastewater effluents from five hospitals (Hospitals A–E) and a commercial facility.

Classification	Antimicrobials	Wastewater ID					
		Commercial Facility	Hospital A	Hospital B	Hospital C	Hospital D	Hospital E
*β*-lactams	Ampicillin	14,175 (N.A.)	27,584 (60,291)	24,889 (22,724)	48,135 (42,096)	10,083 (10,407)	40,622 (57,156)
	Benzylpenicillin	N.D.	N.D.	N.D.	N.D.	157,797 (220,683)	N.D.
	Cefdinir	N.D.	N.D.	N.D.	N.D.	N.D.	N.D.
	Cefpodoxime	N.D.	650 (289)	2406 (2265)	5039 (3226)	8623 (16,701)	824 (372)
	Cefpodoxime proxetil	20 (N.A.)	N.D.	N.D.	N.D.	N.D.	N.D.
	Ceftiofur	N.D.	N.D.	N.D.	N.D.	N.D.	N.D.
New quinolones	Ciprofloxacin	14 (N.A.)	N.D.	134 (115)	389 (251)	184 (142)	105 (16)
	Enrofloxacin	N.D.	N.D.	N.D.	N.D.	N.D.	N.D.
	Levofloxacin	4815 (7547)	9632 (22,272)	5828 (4883)	8752 (10,153)	8296 (12,049)	29,475 (47,798)
Macrolides	Azithromycin	3531 (N.A.)	22,722 (42,593)	1376 (1605)	436 (60)	2268 (1923)	289 (129)
	Clarithromycin	4737 (6827)	3431 (8907)	2635 (3663)	2832 (1986)	1423 (2447)	1823 (2902)
Tetracyclines	Chlortetracycline	N.D.	N.D.	N.D.	N.D.	N.D.	N.D.
	Doxycycline	113 (N.A.)	N.D.	763 (497)	N.D.	39 (N.A.)	723 (N.A.)
	Minocycline	447 (168)	345 (506)	272 (5)	905 (398)	N.D.	N.D.
	Oxytetracycline	38 (N.A.)	N.D.	N.D.	N.D.	N.D.	N.D.
	Tetracycline	114 (N.A.)	795 (739)	N.D.	N.D.	N.D.	N.D.
Glycopeptide	Vancomycin	302 (N.A.)	7257 (5447)	7933 (4806)	31,994 (32,731)	4737 (5129)	10,990 (9676)

## Data Availability

No new data were created or analyzed in this study. Data sharing is not applicable to this article.

## Supplementary Materials

The following supporting information can be downloaded at: https://www.mdpi.com/article/10.3390/antibiotics15010050/s1, Table S1: LC-MS/MS parameters for the validation of each antimicrobial. Table S2: Temporal variations in the ratio of clarithromycin, levofloxacin, and vancomycin in wastewater from hospitals compared to a commercial facility.
