# Supplementary material for: First Multi-Facility Antimicrobial Surveillance in Japanese Hospital Wastewater Reveals Spatiotemporal Trends and Source-Specific Environmental Loads
Source: Antibiotics (Basel). 2026 Jan 3;15(1):50. doi: 10.3390/antibiotics15010050 (PMC12837197; doi:10.3390/antibiotics15010050)
Supplement: Supplementary file 1 [file antibiotics-15-00050-s001.zip › antibiotics-4007107-supplementary.pdf]

Supplemental Information for

First Multi-Facility Antimicrobial Surveillance in Japanese Hospital Wastewater Reveals Spatiotemporal Trends and Source-Specific Environmental Loads

Author names: Takashi Azuma [1,2,*], Ai Tsukada [3], Naoki Fujii [3], Miwa Katagiri [4], Itaru Nakamura [5,6], Hidefumi Shimizu [7], Keita Tatsuno [8], Manabu Watanabe [4], Norio Ohmagari [3,9], Nobuaki Matsunaga [3,9,*]

Affiliation:

[1] Department of Pharmacy, Osaka Medical and Pharmaceutical University, Takatsuki 569-1094, Japan

[2] Center for Infectious Disease Education and Research (CiDER), Osaka University, Suita 565-0871, Japan

[3] AMR Clinical Reference Center, National Center for Global Health and Medicine, Japan Institute for Health Security, Tokyo 162-8655, Japan

[4] Department of Surgery, Toho University Ohashi Medical Center, Tokyo 153-8515, Japan

[5] Department of Infection Prevention and Control, Tokyo Medical University Hospital, Tokyo 160-0023, Japan

[6] Department of Clinical Infectious Diseases, Tokyo Medical University Hospital, Tokyo 160-0023, Japan

[7] Department of Respiratory Medicine, Japan Community Healthcare Organization Tokyo Shinjuku Medical Center, Tokyo 162-8543, Japan

[8] Department of Infection Control and Prevention, Mitsui Memorial Hospital, Tokyo 101-8643, Japan

[9] Disease Control and Prevention Center, National Center for Global Health and Medicine, Japan Institute for Health Security, Tokyo 162-8655, Japan

*Corresponding author:

Takashi Azuma

Affiliation: Department of Pharmacy, Osaka Medical and Pharmaceutical University, 4-20-1 Nasahara, Takatsuki, Osaka 569-1094, Japan, Center for Infectious Disease Education and Research (CiDER), Osaka University, Suita 565-0871, Japan

Tel: +81-72-690-1055, Fax: +81-72-690-1055

e-mail address: takashi.azuma@ompu.ac.jp, t.azuma.mail@gmail.com

Nobuaki Matsunaga

Affiliation: AMR Clinical Reference Center, National Center for Global Health and Medicine, Japan Institute for Health Security, Tokyo 162-8655, Japan, Disease Control and Prevention Center, National Center for Global Health and Medicine, Japan Institute for Health Security, Tokyo 162-8655, Japan

Tel: +81-3-6228-0062, Fax: +81-3-6228-0062

e-mail address: matsunaga.no@jihs.go.jp

Table S1. LC-MS/MS parameters for the validation of each antimicrobial.

Classification	Compound	Ionization mode	Precursor ion (*m/z*)	Product ion (*m/z*)	Cone voltage (V)	Collision energy (eV)	Recovery (%) (SD)	LOD (ng/L)	LOQ (ng/L)
β-lactams	Ampicillin	ESI+	350.2	*105.9*, 192.0	29	24	79 (30)	3.2	10.7
	Benzylpenicillin	ESI+	335.2	*160.1*, 174.0	32	23	85 (17)	1.0	3.3
	Cefdinir	ESI+	369.2	170.0, *227.0*	30	20	91 (13)	0.5	1.6
	Cefpodoxime	ESI+	427.8	*240.5*, 395.9	31	15	81 (20)	0.6	2.1
	Cefpodoxime proxetil	ESI+	557.5	*409.8*, 525.2	30	18	85 (22)	2.4	7.9
	Ceftiofur	ESI+	524.1	*240.9*	38	18	94 (14)	1.3	4.3
New quinolones	Ciprofloxacin	ESI+	332.2	*288.2*, 314.2	40	25	67 (35)	1.3	4.5
	Enrofloxacin	ESI+	360.0	*316.2*, 245.2	37	19	78 (38)	0.5	1.5
	Levofloxacin	ESI+	362.2	261.2, *318.2*	40	21	66 (27)	0.6	2.0
Macrolides	Azithromycin	ESI+	350.2	*105.9*, 192.0	29	24	80 (23)	0.6	2.1
	Clarithromycin	ESI+	748.2	316.6, *558.3*	38	18	88 (18)	0.9	3.1
Tetracyclines	Chlortetracycline	ESI+	479.2	443.5, *461.5*	36	20	91 (14)	0.6	2.1
	Doxycycline	ESI+	445.2	*428.3*	32	18	98 (13)	0.6	2.0
	Minocycline	ESI+	458.3	*441.0*	36	21	124 (12)	0.6	2.0
	Oxytetracycline	ESI+	461.2	*425.8*	28	19	80 (20)	0.6	2.1
	Tetracycline	ESI+	445.2	*409.9*, 427.1	28	20	66 (15)	0.8	2.5
Glycopeptide	Vancomycin	ESI+	724.2	82.9, *100.2*	17	18	92 (14)	1.1	3.8

Product ions in italics were used for quantification

Table S2. Temporal variations in the ratio of clarithromycin, levofloxacin, and vancomycin in wastewater from hospitals compared to a commercial facility.

A)

Sampling date	Ratio of the hospital to the commercial facility					Log$_{10}$ value				
	Hospital A	Hospital B	Hospital C	Hospital D	Hospital E	Hospital A	Hospital B	Hospital C	Hospital D	Hospital E
2023/12/19	0.0	0.1	0.5	1.4	1.8	–1.352	–0.853	–0.311	0.155	0.257
2024/1/9	N.A.	N.A.	0.9	0.0	0.1	N.A.	N.A.	–0.043	–1.334	-1.137
2024/1/16	0.0	0.4	0.8	0.1	0.1	–1.499	–0.353	–0.123	–1.278	-1.082
2024/1/22	0.0	0.3	1.0	0.2	0.3	–1.716	–0.491	–0.020	–0.718	-0.586
2024/1/30	5.3	0.1	0.4	0.1	0.0	0.723	–0.957	–0.419	–1.295	-1.521
2024/2/13	0.1	N.A.	1.3	N.A.	N.A.	–0.824	N.A.	0.112	N.A.	N.A.
2024/2/20	0.0	N.A.	0.3	0.3	0.6	–1.888	N.A.	–0.521	–0.560	-0.258
2024/2/27	0.0	2.1	0.1	N.A.	0.2	–1.320	0.318	–0.926	N.A.	-0.680
2024/3/5	0.1	0.2	0.1	0.0	0.0	–0.978	–0.728	–1.028	–1.620	-1.764
Mean	0.7	0.5	0.6	0.3	0.4	-1.107	–0.511	–0.364	–0.950	–0.846
SD	1.8	0.8	0.4	0.5	0.6	0.818	0.464	0.402	0.612	0.665

B)

Sampling date	Ratio of the hospital to the commercial facility					Log$_{10}$ value				
	Hospital A	Hospital B	Hospital C	Hospital D	Hospital E	Hospital A	Hospital B	Hospital C	Hospital D	Hospital E
2023/12/19	0.1	1.1	0.9	7.0	1.0	–1.029	0.044	–0.032	0.846	0.018
2024/1/9	1.3	0.8	0.3	0.3	0.4	0.116	–0.083	–0.586	–0.527	-0.426
2024/1/16	0.6	0.1	0.6	0.6	1.0	–0.188	–0.864	–0.188	–0.207	-0.012
2024/1/22	0.3	2.6	4.1	0.1	19.8	–0.495	0.421	0.614	–0.986	1.298
2024/1/30	0.6	2.4	1.2	0.3	1.6	–0.211	0.379	0.095	–0.479	0.196
2024/2/13	0.1	0.0	2.1	0.2	1.1	–0.998	–1.492	0.321	–0.642	0.047
2024/2/20	0.2	0.1	0.5	5.2	2.0	–0.668	–1.151	–0.312	0.720	0.291
2024/2/27	0.5	1.8	0.3	0.8	1.6	–0.328	0.265	–0.522	–0.106	0.209
2024/3/5	14.5	2.0	6.6	1.1	27.5	1.162	0.307	0.817	0.053	1.440
Mean	2.0	1.2	1.8	1.8	6.2	–0.293	–0.242	0.023	–0.148	0.340
SD	4.7	1.0	2.1	2.5	10.1	0.664	0.730	0.487	0.611	0.619

C) Vancomycin

Sampling date	Ratio of the hospital to the commercial facility					Log$_{10}$ value				
	Hospital A	Hospital B	Hospital C	Hospital D	Hospital E	Hospital A	Hospital B	Hospital C	Hospital D	Hospital E
2023/12/19	18.9	21.2	15.8	6.0	49.8	1.277	1.326	1.198	0.775	1.697
2024/1/9	19.8	21.2	64.1	12.4	26.0	1.296	1.327	1.807	1.095	1.414
2024/1/16	18.2	32.1	325.0	13.1	21.8	1.261	1.506	2.512	1.117	1.338
2024/1/22	19.7	40.0	205.2	19.2	36.1	1.294	1.602	2.312	1.284	1.558
2024/1/30	55.1	8.1	73.8	13.6	57.8	1.741	0.909	1.868	1.135	1.762
2024/2/13	12.4	6.9	187.1	4.4	10.5	1.093	0.839	2.272	0.647	1.019
2024/2/20	10.8	15.3	25.5	59.1	8.2	1.035	1.185	1.407	1.772	0.915
2024/2/27	54.7	36.9	43.1	4.6	9.9	1.738	1.567	1.635	0.663	0.995
2024/3/5	6.9	54.8	14.6	8.8	107.8	0.838	1.739	1.164	0.942	2.033
Mean	24.0	26.3	106.0	15.7	36.4	1.286	1.333	1.797	1.048	1.415
SD	18.0	15.9	108.5	17.0	32.1	0.299	0.310	0.493	0.351	0.386

N.A.: Not available
